# Magnetic Resonance Imaging and Nuclear Imaging of Parkinsonian Disorders: Where do we go from here?

**DOI:** 10.2174/1570159X21666230801140648

**Published:** 2024-08-01

**Authors:** Félix-Antoine Savoie, David J. Arpin, David E. Vaillancourt

**Affiliations:** 1 Department of Applied Physiology and Kinesiology, Laboratory for Rehabilitation Neuroscience, University of Florida, Gainesville, FL, USA;; 2 Department of Neurology, Fixel Institute for Neurological Diseases, University of Florida, Gainesville, FL, USA;; 3 J. Crayton Pruitt Family Department of Biomedical Engineering, University of Florida, Gainesville, FL, USA

**Keywords:** Parkinson’s disease, magnetic resonance imaging, nuclear imaging, cerebral blood flow, MSA, corticobasal degeneration

## Abstract

Parkinsonian disorders are a heterogeneous group of incurable neurodegenerative diseases that significantly reduce quality of life and constitute a substantial economic burden. Nuclear imaging (NI) and magnetic resonance imaging (MRI) have played and continue to play a key role in research aimed at understanding and monitoring these disorders. MRI is cheaper, more accessible, non-irradiating, and better at measuring biological structures and hemodynamics than NI. NI, on the other hand, can track molecular processes, which may be crucial for the development of efficient disease-modifying therapies. Given the strengths and weaknesses of NI and MRI, how can they best be applied to Parkinsonism research going forward? This review aims to examine the effectiveness of NI and MRI in three areas of Parkinsonism research (differential diagnosis, prodromal disease identification, and disease monitoring) to highlight where they can be most impactful. Based on the available literature, MRI can assist with differential diagnosis, prodromal disease identification, and disease monitoring as well as NI. However, more work is needed, to confirm the value of MRI for monitoring prodromal disease and predicting phenoconversion. Although NI can complement or be a substitute for MRI in all the areas covered in this review, we believe that its most meaningful impact will emerge once reliable Parkinsonian proteinopathy tracers become available. Future work in tracer development and high-field imaging will continue to influence the landscape for NI and MRI.

## 1 INTRODUCTION

Parkinsonian disorders are a heterogeneous group of progressive and incurable neurodegenerative diseases characterized by bradykinesia, resting tremor, rigidity, gait impairment, and long-term cognitive impairment, all of which significantly diminish the quality of life [1]. Although these diseases show distinct patterns of neurodegeneration [2-4], a common denominator is degeneration of the substantia nigra (SN) [2-10], a small cluster of midbrain dopaminergic neurons. Despite not being mutually exclusive [11], Parkinsonian disorders are generally categorized as synucleinopathies or tauopathies depending on whether pathological aggregates of alpha-synuclein (𝛼Syn) or tau proteins are found within the brain post-mortem. Parkinson’s disease (PD), Multiple system atrophy (MSA), and Dementia with Lewy bodies (DLB) fall under the umbrella of synucleinopathies, whereas progressive supranuclear palsy (PSP) and corticobasal degeneration (CBD) are tauopathies [11]. Depending on clinical symptoms, MSA can further be classified as cerebellar (MSA-C, associated with dysarthria and ataxia) or Parkinsonian (MSA-P, associated with bradykinesia and tremor) [12]. Distinct clinical phenotypes also exist for PSP, such as PSP-Richardson’s syndrome (PSP-RS) and PSP-parkinsonism (PSP-P) [13]. PD is by far the most prevalent Parkinsonian disorder, with an age-standardized rate of ~109 cases per 100,000 people globally [14]. Although variability exists between studies, the annual incidence rate of PD (~10-20 per 100,000 person-years [14-17]) is estimated to be 2-4 times greater than PSP (~5.3 per 100,000 person-years [18]), 3-6 times greater than both DLB (~3.5 per 100,000 person-years [15]) and MSA (~3.0 per 100,000 person-years [18]), and more than 10 times greater than that of CBD (~0.62-0.92 per 100,000 person-years [19]). Despite being less common than PD, it is worth noting that MSA, PSP, and CBD are generally associated with worse prognoses, with the average patient succumbing ~5-9 years after disease onset [12, 18, 20-23].

The goals of research on Parkinsonian disorders are to identify treatments that can reverse (or at least slow down) the pathological processes at play and strategies that can prevent the disease from taking root in the first place. To achieve this, tools that provide a window through which Parkinsonian disorders can be understood and monitored are needed. Currently, rating scales such as the Movement Disorders Society's Unified Parkinson's Disease Rating Scale (MDS-UPDRS, [24]), the Unified Multiple System Atrophy Rating Scale (UMSARS, [25]), the Progressive Supranuclear Palsy Rating Scale (PSPRS, [26, 27]), and the Cortical Basal ganglia Functional Scale (CBFS, [28]) are the gold standard to assess Parkinsonian disorder severity. Despite their usefulness, these symptom-based ordinal scales cannot distinguish between disease-modifying and symptomatic intervention effects. This disambiguation requires reliable, accessible, cost-efficient, and relevant disease biomarkers. To this end, neuroimaging methods such as single-photon emission computed tomography (SPECT), positron emission tomography (PET), and magnetic resonance imaging (MRI) have become invaluable [11, 29-33]. Both SPECT and PET fall under the category of nuclear imaging (NI), as they both require the injection of a radioactive tracer that binds with a biological molecule of interest. As the tracer decays, it either directly (SPECT) or indirectly (PET) emits photons, which are used to infer the ligand binding site by way of tomographic reconstruction [34, 35]. Despite its usefulness in clinical and research contexts, NI comes with some downsides. The most obvious is that SPECT and PET require the use of radioactive tracers, which, despite being relatively safe in small/infrequent doses, should be monitored closely. Moreover, the relatively short half-lives of PET and SPECT tracers can be significant barriers to their use. Although the longer half-lives of ^123^I- and ^99m^Tc-labelled SPECT tracers (13 and 6 hours, respectively) allow for more latitude, the short half-lives of ^11^C- and ^18^F-labelled PET tracers (20 and 110 min, respectively) requires an on-site cyclotron or local distribution center. In contrast to NI, MRI does not require the injection of a radioactive agent. Moreover, MRI is generally less expensive than NI due to the cost of radiotracers, making it more accessible in clinical and research settings. Given the strengths and weaknesses of each imaging modality, what should be the focus of NI and MRI in the ongoing Parkinsonism research? In this review, we examine the usefulness of NI and MRI in three areas of Parkinsonism research to highlight where each modality can be most impactful. These areas are differential diagnosis, prodromal disease identification, and disease monitoring.

## 2 DIFFERENTIAL DIAGNOSIS

Until an autopsy is performed to confirm the underlying pathology, the diagnosis of a specific Parkinsonian disorder cannot currently be ascertained. The opinion of expert physicians, which largely relies on clinical symptoms and medical history [36-39], is currently considered the gold standard for antemortem diagnosis. Expert opinion, however, is not infallible. Indeed, the differential diagnosis of Parkinsonian disorders has proven challenging [40-44], especially in early-stage disease, because of overlapping motor and non-motor manifestations. For instance, Hughes *et al.* [40] reported that 24% of PD diagnoses are incorrect, with about half of the misdiagnoses being PSP or MSA. Likewise, a retrospective pathological study of 134 patients with an antemortem MSA diagnosis reported a misdiagnosis rate of 38%, 82% of which were reclassified as DLB (37%), PSP (29%), or PD (16%) [42]. Not only is an accurate differential diagnosis important for the counseling and management of patients in clinical settings, but it is also important for the categorization of patients in clinical trials and research. It follows that imaging biomarkers that could assist with differential diagnosis would have great value in clinical and research settings. In the following section, we review the NI and MRI methodologies that have proven valuable for differential diagnosis. The diagnosis ground truth cited below was clinical expert opinion unless otherwise stated.

### 2.1 Metabolic Imaging

^18^F-fluorodeoxyglucose (^18^F-FDG) PET is appealing to study neurological diseases because abnormal brain metabolism is indicative of brain dysfunction, which is the root cause of disease symptoms. Because Parkinsonian disorders are characterized by different symptoms, they alter cerebral glucose metabolism in different ways [45]. In their 2017 meta-analysis, Meyer *et al.* [46] reported that ^18^F-FDG PET readings supported by voxel-wise statistical analyses could reliably differentiate PD from atypical Parkinsonism with high sensitivity and specificity (both > 90%). A similar approach was also shown to distinguish between MSA, PSP, and CBD with high specificity (≥ 90%) and moderate to high sensitivity (74-100%) [45, 47-49]. In a recent retrospective study, Caminiti *et al.* [50] used the ^18^F-FDG PET scans of 70 atypical Parkinsonian patients, many of whom had uncertain clinical diagnoses at the moment of scanning, to establish a probable diagnosis. The ^18^F-FDG PET-based diagnoses were then compared to the diagnoses established during a follow-up as the standard of truth. The authors found that their approach differentiated between PSP, MSA-C, DLB, and CBD with 99% accuracy. They further reported four cases where the first clinical diagnosis differed from that predicted by ^18^F-FDG PET but was changed to that predicted by ^18^F-FDG PET at follow-up. Spatially covariant ^18^F-FDG uptake patterns (rather than regional differences in ^18^F-FDG uptake) can also assist with disease differentiation. For instance, Tang *et al.* [51] showed that the PD-related pattern (PDRP) can distinguish PD from MSA and PSP with high sensitivity (84%) and specificity (97%). Likewise, they reported that the MSA-related pattern distinguished MSA from PD and PSP (sensitivity: 85%; specificity: 96%) and that the PSP-related pattern distinguished PSP from PD and MSA (sensitivity: 88%; specificity: 94%). These findings have been qualitatively confirmed in a separate cohort [52]. Together, these results highlight the usefulness of metabolic PET for the differentiation of Parkinsonian disorders.

Abnormal patterns of cerebral blood flow (CBF), a correlate of metabolic activity that can be measured with lipophilic NI markers such as [^99m^Tc]-labelled ethyl cysteine dimer and hexamethyl propylene amine oxime [53], have been observed in Parkinsonian disorders [54-60]. Some of these patterns may help differentiate between disorders. For instance, Kimura *et al.* [58] reported that thalamic hypoperfusion accurately differentiates PSP from PD (sensitivity: 89%; specificity: 84%) and MSA-P (sensitivity: 92%; specificity: 79%) and that cerebellar hypoperfusion respectably differentiates MSA-P from PD (sensitivity: 86%; specificity: 67%, see also [59]). Despite these findings, it is difficult to assess how reliably perfusion NI can differentiate between Parkinsonian disorders, given the paucity of published literature on this topic.

### 2.2 Structural MR

**Fig. (1):**
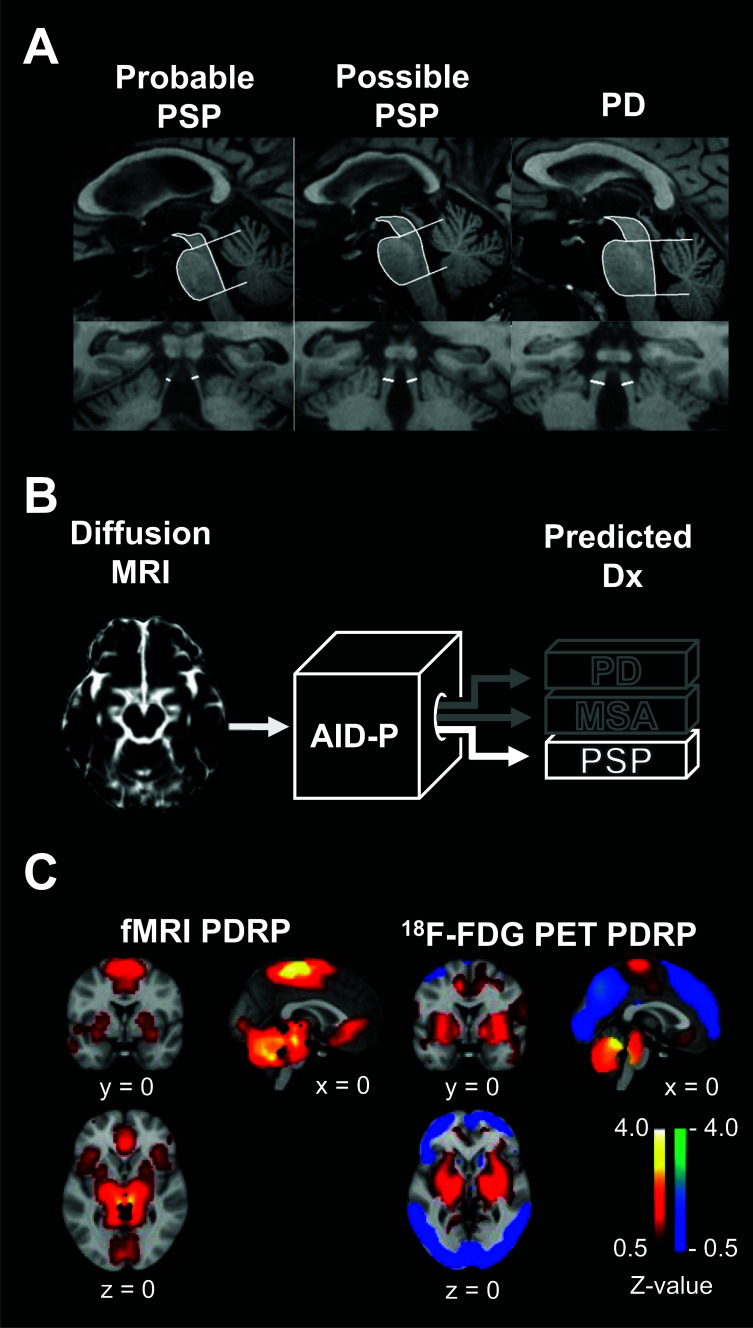
(**A**) T1-weighted images show greater atrophy of the midbrain (top row) and superior cerebellar peduncles (bottom row) in probable PSP (left) and possible PSP (middle) compared to PD (right). The MRPI uses these distinctive morphological differences to differentiate PSP from other forms of Parkinsonism (adapted from Nigro *et al.* [267]). (**B**) Conceptual representation of the AID-P [78]. The AID-P extracts measures from diffusion MRI scans obtained from patients with Parkinsonism and uses a support vector machine to predict diagnosis. (**C**) PDRP obtained from resting-state fMRI (left) and metabolic PET (right) (adapted from Vo *et al.* [102]). Notice the overlap between the fMRI PDRP and the areas showing increased metabolism in the ^18^F-FDG PET PDRP. Abbreviations: MRPI, magnetic resonance Parkinsonism index; PD, Parkinson's disease; PSP, progressive supranuclear palsy; MRI, magnetic resonance imaging; AID-P, automated imaging differentiation in Parkinsonism; Dx, Diagnosis; MSA, multiple system atrophy; fMRI, functional magnetic resonance imaging; PDRP, Parkinson’s disease-related pattern; FDG, fluorodeoxyglucose; PET, positron emission tomography.

T1-weighted MRI can be used to reveal disease-specific structural brain anomalies, which can assist with differential diagnosis. For instance, PSP patients show significant midbrain and superior cerebellar peduncle atrophy compared to other forms of Parkinsonism [61-64] (Fig. **1A**). As such, PSP can be differentiated from PD (sensitivity: 80%; specificity: 90%) and MSA-P (sensitivity: 79%; specificity: 92%) based on the sagittal midbrain area [62]. Quattrone *et al.* [61] combined the pons/midbrain and medium/superior cerebellar peduncle ratios into a metric called magnetic resonance Parkinsonism index (MRPI), which differentiated PSP from PD, MSA-P, and healthy controls (HC) with 100% accuracy. Subsequent studies have confirmed the value of the MRPI for differentiating PSP from PD, MSA, and CBD, albeit with slightly less accuracy [65-67]. A recently published study conducted on a large international cohort has shown the MRPI to robustly differentiate both PSP-RS and PSP-P from non-PSP individuals (*i.e.*, PD, MSA, and HC) with an accuracy > 85% [63]. In addition to the midbrain and peduncular atrophy, PSP patients often present with an enlarged third ventricle [68, 69], which can further help with differential diagnosis. In a recent international study, Quattrone *et al.* [68] showed that the ratio between the third ventricle width normalized by the internal skull diameter differentiates early-stage PSP from *de novo* PD patients with an accuracy of 87%. The MRPI 2.0, which combines the MRPI with the width of the third ventricle and frontal horns, was significantly better than MRPI at differentiating PSP-P from PD despite that the PSP-P patients had not yet developed vertical supranuclear gaze palsy, the most specific sign of this disease [69]. Moreover, the MRPI 2.0 differentiates patients initially diagnosed with PD as having PSP four years before their diagnosis was changed due to the development of vertical gaze abnormalities with 100% accuracy [70]. Overall, the above-mentioned studies suggest that simple structural MRI metrics such as the MRPI and MRPI 2.0 can reliably distinguish PSP from other forms of Parkinsonism.

Despite its value for the diagnosis of PSP, the MRPI does not perform well to differentiate MSA-P from either PD (sensitivity: 56%; specificity: 89%) or CBD (sensitivity: 100%; specificity: 63%), nor PD from CBD [66]. These shortcomings can be overcome by looking at structures other than the midbrain, cerebellar peduncles, or third ventricle. For instance, Chougar *et al.* [71] compared 4 supervised machine learning approaches (linear regression, support vector machine (SVM) with either a linear or radial basis kernel, and random forest) for differentiating PD, MSA-P, MSA-C, and PSP based on the volumes of 13 segmented brain regions involved in Parkinsonism (*i.e.*, 13 data points per patient). Each volume was corrected for total intracranial volume to account for interindividual variability and normalized to volumes obtained from controls scanned using the same magnet to reduce inter-scanner effects. The algorithms were then trained and validated on a cohort of 63 PD, 11 MSA-P, 12 MSA-C, and 22 PSP patients using a five-fold cross-validation procedure carried out on 50 stratified random splits. Of the 50 models generated per machine learning approach, the one with the highest balanced accuracy (and an appearance frequency > 10%) was selected and tested on a separate cohort (PD = 56; MSA-P = 24; MSA-C = 11; PSP = 30). Overall, linear regression, linear SVM, and random forest all performed similarly and outperformed radial basis SVM. Specifically, linear regression, linear SVM, and random forest separated PD from MSA-P with ≥ 60% accuracy (although note that linear regression and SVM were 77% and 80% accurate, respectively) and from MSA-C with ≥ 91% accuracy. These algorithms also differentiated PSP from PD (≥ 84% accuracy), MSA-P (≥ 80% accuracy), and MSA-C (≥ 97% accuracy). MSA-P was differentiated from MSA-C with ≥ 71% accuracy. In another study, Constantinides *et al.* [66] made midbrain, pons, and corpus callosum planimetry measurements in Parkinsonian patients (*i.e*., PD, MSA, PSP, and CBD) and healthy controls. The authors reported that the MRPI differentiated PSP from every other patient group with relatively high sensitivity (73-100%) and specificity (all 100%). They also reported that the posteroanterior callosal gradient, defined as the measured midsagittal surface difference between the anterior corpus callosum (CC_1_) and the sum of its posterior parts (*i.e.*, CC_2_+CC_3_+CC_4_+CC_5_, see [72]) differentiated CBD from PD (sensitivity: 75%; specificity: 100%) and MSA-P (sensitivity: 100%; specificity: 75%). Although these results need to be replicated, they strongly suggest that structural MRI may be able to distinguish between PD, MSA, PSP, and CBD.

### 2.3 Diffusion MRI

Diffusion-based imaging can be used to investigate tissue microstructure at the voxel-level. Hence, much like structural MRI, it can be used to detect disease-specific anomalies that can assist with differential diagnosis. Specifically, the development of multi-compartment diffusion models [73, 74], which separate unrestricted (free-water) from restricted (tissue) diffusion, has allowed researchers to make inferences about brain tissue integrity. For instance, free-water (FW) is expected to increase with atrophy-based neurodegeneration and neuroinflammation [75, 76]. In support, numerous studies have reported that FW is elevated in the SN and other brain regions of patients affected by Parkinsonian disorders [77-87] and that posterior SN FW inversely correlates with NI-derived measures of dopamine function [82-85]. Despite this association, SN FW and NI-derived measures of dopamine function are not redundant measures. Indeed, using backward stepwise linear regression, Yang *et al.* [84] showed that SN FW and striatal VMAT2 PET binding are complementary predictors of Hoehn & Yahr stage, MDS-UPDRS part III, posture, gait, and dementia ratings in early-stage PD. The fact that PD-related differences in SN FW are not necessarily accompanied by differences in voxel-based morphometry also suggests that FW is not merely a redundant measure of structural atrophy [82, 83].

FW and FW-corrected diffusion measures have proven useful for the differential diagnosis of Parkinsonian disorders. Planetta *et al.* [77] reported FW and corrected fractional anisotropy (FA_T_) differences between PD, MSA, PSP, and HC in several brain regions known to be affected in Parkinsonism. The authors later showed that a SVM could use the extracted FW and FA_T_ measures to categorize patients with high sensitivity (> 93%) and specificity (> 83%). These findings paved the way for a large multicenter study by Archer *et al.* [78], who developed an automated diffusion imaging-based approach for differentiating Parkinsonian disorders (the automated imaging differentiation in Parkinsonism, AID-P, Fig. **1B**). The AID-P requires FW and FA_T_ values from 17 subcortical brain regions and 43 known white matter tracts (*i.e.*, 120 data points per patient), which are automatically extracted from standard diffusion-weighted MRI scans. For classification, it relies on SVM-derived models trained and validated (using a five-fold cross-validation procedure) to distinguish between PD and atypical parkinsonism (training cohort: PD = 406; MSA = 70; PSP = 103) and between MSA and PSP (training cohort: MSA = 71; PSP = 99). In the PD *vs.* atypical parkinsonism test cohort (PD = 105, MSA = 14; PSP = 26), the AID-P distinguished PD from MSA and PSP with 90% accuracy. In the MSA *vs.* PSP test cohort (MSA = 13, PSP = 30), it classified patients with 82% accuracy, making the AID-P a convenient two-step process for the differentiation of Parkinsonian disorders. Although anecdotal, Archer and colleagues were able to confirm the diagnosis of five patients with post-mortem autopsies. These autopsies revealed that the AID-P had correctly classified all five of these patients, even though one PSP patient had been clinically misdiagnosed as MSA before death. These results suggest that like structural MRI, diffusion-based MRI can non-invasively assist with the differential diagnosis of PD, MSA, and PSP.

Diffusion MRI studies have also highlighted differences between CBD and other Parkinsonian disorders [88-92], and these have shown promise for differential diagnosis. For instance, Rizzo *et al.* [90] reported that apparent diffusion coefficient asymmetry between the left and right hemispheres could distinguish CBD from both PD and PSP with 100% accuracy. In another study, Boelmans *et al.* [88] reported that the mean diffusivity of the corpus callosum differentiated CBD from PD with a sensitivity and specificity of 79%. It should be mentioned, however, that these studies did not use FW-corrected diffusion metrics. Hence, it is unclear if the anomalies identified in the CBD patients reflected changes in tissue microstructure or extracellular FW [73]. Future studies should investigate whether FW-corrected diffusion metrics can differentiate CBD patients from other forms of Parkinsonism.

### 2.4 Functional MRI (fMRI)

Like metabolic PET, functional MRI (fMRI) provides a window into brain function/dysfunction because the measured blood-oxygen-level-dependent (bold) signal is driven by neural activity [93]. Indeed, fMRI can be used to highlight temporally coherent brain networks (functional connectivity), as well as brain regions that are engaged during motor and cognitive tasks performed in the scanner. Many studies have reported that Parkinsonian disorders disturb normal resting-state functional connectivity [94-101]. However, few have investigated whether resting-state functional connectivity can differentiate between Parkinsonian disorders. In one study, Baggio *et al.* [94] reported that cerebellar functional connectivity could be used to distinguish PD from MSA with 77% accuracy, suggesting that functional connectivity differences could assist with differential diagnosis. Recently, it was shown that a PDRP can be obtained from resting-state fMRI data using a combination of independent component analysis, dual regression, and bootstrapping [102, 103]. This fMRI PDRP is characterized by increased activity in the basal ganglia, cerebellum/pons, thalamus, anterior cingulate cortex, and supplementary motor area, which is consistent with the regions showing increased ^18^F-FDG uptake in PD (Fig. **1C**) [104]. Moreover, expression of the fMRI PDRP is correlated with motor disability ratings [102, 103]. Given these findings, perhaps resting-state fMRI, like ^18^F-FDG PET, could be used to identify bold covariance patterns specific to MSA, PSP, and CBD. In this case, resting-state fMRI could provide a non-invasive analogue to ^18^F-FDG PET for differential diagnosis.

Task-based fMRI studies have also reported bold response differences between Parkinsonian disorders [105-107]. For instance, it was shown that bold responses in numerous visuomotor areas differ between PSP and both PD and MSA patients during a manual force-matching task [105, 106]. Whether such differences can differentiate between Parkinsonian disorders, however, has not yet been demonstrated. Because task-based fMRI provides static beta coefficient maps, PCA-based approaches such as the scaled subprofile model [45, 104, 108] could be used to identify task-related covariance patterns specific to PD, MSA, PSP, and CBD. These, in turn, could potentially help with differential diagnosis. Moreover, because the tasks performed in the scanner can be tailored to engage neural circuits involved in disease-specific symptoms (*e.g.*, oculomotor dysfunction in PSP), task-based fMRI could be more revealing than passive imaging. More research is needed in this space.

Another fMRI technique, arterial spin labelling (ASL), tracks the movement of arterial water [109, 110]. Although both ASL and bold imaging depend on hemodynamics, ASL may provide more spatially precise estimates of metabolic activity because it can provide an isolated measure of CBF (unlike bold, which conflates CBF, cerebral blood volume, and oxygen consumption [109, 110]). ASL is also an appealing alternative to perfusion NI for reasons alluded in the introduction. Several ASL studies have highlighted abnormal patterns of CBF in Parkinsonian disorders that could be useful for their differential diagnosis [111-118]). Few studies, however, have evaluated the value of ASL-based CBF measures for distinguishing between Parkinsonian disorders [112-114]. In one of the studies, Cui *et al.* [113] reported that cerebellar perfusion discriminates MSA-P from MSA-C with high accuracy (specificity: 100%; sensitivity: 79%; area under the curve: 94%). In another study, Cheng *et al.* [112] reported that PD could be differentiated from PSP based on CBF differences in the left SMA or right thalamus with fair accuracy (sensitivity: 75-80%; specificity: 75-81%). Although these studies highlight the potential value of ASL for differentiating certain Parkinsonian disorders, more studies with larger cohorts are to be required to confirm which regional CBF measures accurately distinguish the disorders. Multimodal imaging studies that directly compare ASL and perfusion NI in Parkinsonian disorders would also help establish the interchangeability of both methods (*e.g.*, [119]).

Concomitant fMRI and electromyography (EMG) studies have uncovered correlates of PD resting tremor. According to the “dimmer-switch” hypothesis, PD resting tremor results from the interplay between the basal ganglia and cerebello-thalamo-cortical motor loops, both of which share common motor cortical nodes [120]. In this model, the basal ganglia dictate changes in tremor amplitude by modulating cerebello-thalamo-cortical motor loop activity [121, 122]. Tremor is also frequently seen in MSA and, to a lesser extent, in PSP [123-125], but it is unclear if the underlying mechanisms are the same as in PD. Interestingly, some evidence suggest that tremor characteristics differ between PD and atypical Parkinsonian disorders [124-126]. For example, whereas PD resting tremor often shows harmonics and manifests with a “pill-rolling” motion, MSA tremor does not [124, 126]. This raises the possibility that PD, MSA, and PSP tremors are subtended by distinct network anomalies. Identifying and differentiating these tremor networks with fMRI could be proved beneficial for the differential diagnosis of Parkinsonian disorders.

### 2.5 Interim Summary

Metabolic PET, diffusion-weighted MRI, and structural MRI have proven similarly valuable for the differentiation of Parkinsonian disorders. Although few studies have evaluated the value of fMRI for differential diagnosis, recent findings indicate that this type of imaging has potential. Since MRI is generally more accessible and non-invasive compared to NI, some centers may be more inclined to prefer MRI in future clinical contexts.

## 3 BIOMARKERS OF PRECLINICAL/PRODROMAL PARKINSONISM

Current evidence suggests that Parkinsonian disorders are preceded by a long period during which the disease is either completely asymptomatic (preclinical) or too subtle to be diagnosed (prodromal) [127-131]. This is supported by estimations from pathological studies, which suggest that there is substantial nigral cell loss before the onset of clinical symptoms [5, 8, 132]. If a post-diagnosis time window exists beyond which the brain damage is irremediable, the detection of preclinical Parkinsonism may be the biggest determinant of future treatment success. Therefore, imaging biomarkers that can highlight preclinical Parkinsonian disorders are of paramount importance.

The identification of neuroimaging biomarkers of preclinical/prodromal Parkinsonian disorders requires the study of individuals with an increased risk of developing these diseases in the future. Although asymptomatic carriers of Parkinsonism-related genetic mutations can be studied as a model of preclinical Parkinsonism [133], many carriers will never develop clinical symptoms due to incomplete gene penetrance [134-137]. Moreover, it is unclear whether genetic Parkinsonism mimics idiopathic Parkinsonism, which is far more common [1]. Patients with rapid-eye-movement behavior disorder (RBD), a parasomnia characterized by abnormal dream-enactment, currently provide the best opportunity to investigate prodromal Parkinsonism [138, 139]. In a recent systematic review and meta-analysis, Galbiati, *et al.* [140] estimated that > 90% of all RBD patients would develop a neurodegenerative disease within 14 years, with most of these being synucleinopathies (see also [141]). Indeed, ~32% of all the RBD patients included in their systematic review (*i.e.*, 1235/3865) converted into a neurodegenerative disease after a mean follow-up of ~5 years, with 74% of them developing a synucleinopathy (PD = 44%; DLB = 25%; or MSA = 5%). This, combined with clinicopathological data highlighting the high frequency of synucleinopathy in RBD patients [142], clearly suggests that RBD is a key prodromal model of PD, DLB, and MSA. Here, we thus focus on imaging findings in RBD patients.

### 3.1 Dopaminergic Imaging

**Fig. (2):**
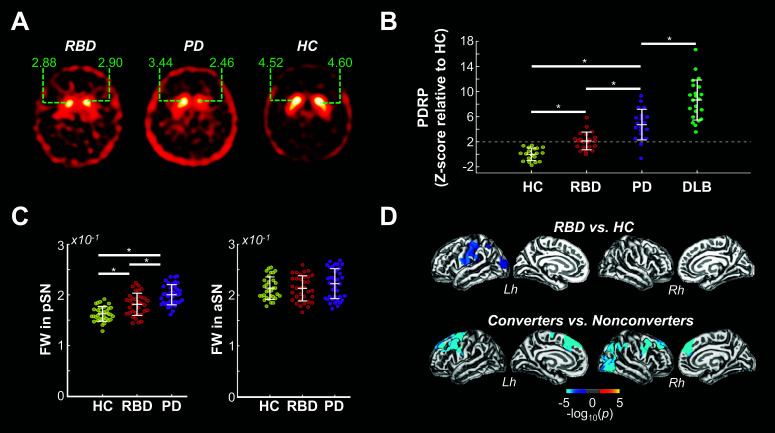
(**A**) Reduced striatal binding ratios (green) in RBD (left) and PD (middle) compared to HC (right) (Eisensehr *et al.* [149]). (**B**) Expression of the PDRP in HC, RBD, PD, and DLB (Meles *et al.* [151]). Notice that PDRP expression in HC < RBD < PD. (**C**) FW values in the pSN (left) and aSN (right) of HC, RBD, and PD patients (Zhou *et al.* [85]). Notice that pSN FW in HC < RBD < PD. (**D**) Cortical areas showing reduced thickness in RBD patients compared to HC (top) and in RBD patients who converted to an overt synucleinopathy after a 3-year follow-up compared to RBD patients who remained disease-free over the same period (bottom) (Pereira *et al.* [209]). **Abbreviations:** HC, healthy controls; RBD, rapid eye movement behaviour disorder; PD, Parkinson’s disease; R, right; L, left; PDRP, Parkinson’s disease-related pattern; DLB, Dementia with Lewy Bodies; FW, free-water; pSN, posterior substantia nigra; aSN anterior substantia nigra; Lh, left hemisphere; Rh, right hemisphere; *p < 0.05.

According to the established criteria [138], an abnormal dopaminergic PET/SPECT scan (*i.e.*, a specific binding ratio of 2 SDs below the mean of healthy controls) is the second strongest indicator of prodromal PD (likelihood ratio = 43.3), trailing only polysomnography-confirmed RBD (likelihood ratio = 130). Unsurprisingly, nigrostriatal dysfunction has frequently been reported in RBD patients [143-158] (Fig. **2A**). For instance, a recent retrospective study has reported abnormal dopamine transporter (DAT) SPECT binding in 73/129 RBD patients [145]. Out of the 44 patients who converted into a neurodegenerative disorder during the study’s timeline, 39 developed a synucleinopathy (PD = 21, DLB = 16; MSA = 2). Interestingly, the patients who converted within 5 years of scanning (N = 34) showed significantly lesser striatal binding ratios compared to those who converted later or remained disease-free, suggesting that DAT binding is a predictor of short-term phenoconversion in RBD patients [144, 150]. In another retrospective study of 263 RBD patients Arnaldi, *et al.* [143] reported that ~ 20% of patients converted to an overt synucleinopathy within 2 years (PD = 33; DLB = 18; MSA = 1). The authors reported that, among other clinical variables, abnormal striatal DAT SPECT binding was the strongest predictor of short-term phenoconversion (hazard ratio of 4.35). Overall, these studies suggest that dopaminergic imaging is a valuable biomarker of prodromal synucleinopathy and that DAT imaging is a predictor of short-term phenoconversion.

### 3.2 Metabolic Imaging

Like dopaminergic imaging, metabolic imaging has highlighted metabolic anomalies in RBD patients. Specifically, ^18^F-FDG PET studies have shown that RBD patients express the PDRP to a significantly greater degree than HC [151, 159-161] (Fig. **2B**), and some data suggests that higher PDRP scores are associated with an increased risk of short-term phenoconversion [159, 162]. Shin *et al.* [160] have recently shown that RBD patients express a metabolic covariance pattern identified in *de novo* PD patients with a preclinical history of RBD to a greater extent than they do the standard PDRP. They further reported that expression of this pattern within 1.5 and 3.0 standard deviations above the mean of HC significantly predicts short-term conversion (~3 years) in RBD patients. In comparison, PDRP expression significantly predicted conversion only if it was within 1.40 and 1.97 standard deviations above that of the healthy control mean. Although these results need to be independently confirmed, they suggest that ^18^F-FDG PET-derived covariance patterns can be used to identify RBD patients at risk of short-term conversion for enrollment in clinical trials.

Like ^18^F-FDG PET, perfusion SPECT has revealed metabolic anomalies in RBD patients compared to healthy controls. Specifically, reduced CBF has been reported in all cerebral cortices, as well as the cerebellum, pons, insula, uncus, and cingulate cortex [163-168]. Some studies have also reported increased perfusion in the cerebellum, pons, hippocampus, parahippocampal cortex, putamen, globus pallidus internus, and paracentral cortex [159, 164, 167-169]. Although some of these contradictory findings are puzzling, hyperperfusion may be a transient compensatory mechanism that eventually fails with disease progression. Another possibility is that increased CBF in RBD patients is an artifact of global CBF normalization, which can invert *true* perfusion differences if global CBF is not equivalent between groups [54]. In any case, it appears that perfusion NI can detect differences between RBD patients and healthy controls, which suggests that it could help identify prodromal synucleinopathy. Evidence also suggests that the PDRP, which can be calculated from perfusion NI, may be able to predict short-term phenoconversion when combined with age [159].

### 3.3 Iron-sensitive MRI

Abnormal iron homeostasis in the brain has been suggested to play a key role in the pathogenesis of neurodegenerative disorders such as PD [170, 171]. Because brain iron is generally paramagnetic, it increases magnetic susceptibility and can be measured with MRI [172-174]. Indeed, paramagnetic sources within tissues quicken transverse relaxation by accelerating the rate at which precessing protons fall out of phase. The resulting signal characteristics can be used to infer the presence of iron deposits using methods such as R2* relaxometry, susceptibility-weighted imaging (SWI), and quantitative susceptibility mapping (QSM) [175-177]. Whereas R2* and SWI highlight magnetic field variations due to magnetic susceptibilities, QSM quantifies the magnetic susceptibilities that cause the magnetic field variations [173]. This provides QSM with at least two advantages over R2* and SWI for brain imaging. First, QSM can remove geometry- and orientation-dependent blooming artifacts, which can yield more spatially accurate estimates of iron deposits [173]. Second, QSM can account for the masking effects of water, which tends to decrease the magnetic susceptibility of tissue because it is diamagnetic [174]. This is particularly important because the subcortical areas prone to iron accumulation in Parkinsonian disorders often show increased FW levels [77].

Studies using iron-sensitive MRI have generally shown increased SN iron content in PD [178], with SWI and QSM values positively correlating with UPDRS scores. Iron-sensitive MRI has also revealed increased subcortical iron in atypical Parkinsonism [179]. In RBD patients, some studies have found abnormal iron accumulation in the basal ganglia [152, 180-182], while others have not [158, 183, 184]. Although low statistical power [183, 184] and qualitative image evaluation [152, 180] can explain these discordant findings, two of the three studies that reported null findings used R2* relaxometry, which may be less robust than QSM for reasons stated above. For instance, Sun *et al.* [181] reported that SN QSM values in RBD patients were greater than in HC but lesser than in PD, consistent with the idea that RBD shows SN changes before developing a type of Parkinsonism. Although SN QSM values only differentiated RBD from HC with low/moderate sensitivity (68-76%) and specificity (66-80%), the findings of Sun *et al.* [181] suggest that QSM of the SN has some potential as a prodromal synucleinopathy marker.

### 3.4 Neuromelanin-sensitive MRI

Catecholaminergic neurons oxidize excessive dopamine (DA) and norepinephrine into quinones [185], which agglomerate with other molecules within autophagic lysosomes to form dark pigments called neuromelanin (NM) [185]. Unless cell death causes NM to leak into the extracellular space where it is eliminated by microglia [186], it typically accumulates within catecholaminergic neurons throughout life [187, 188]. This is not the case in PD patients, who show reduced SN NM concentrations compared to age-matched HC due to DA cell loss [187]. NM can be detected using MRI because it acts as a cytosolic metal chelator, which makes it highly paramagnetic [185]. Indeed, several studies have provided evidence for diminished NM in the SN and/or locus coeruleus (LC) of individuals with Parkinsonian disorders [157, 158, 189-193], and a positive correlation has been shown between signal intensity in NM-sensitive MRI and NM-containing neuron density in the SN [194]. Quantitative [184] and qualitative [190] evidence suggests that SN NM volume is reduced in RBD patients, particularly in the posterolateral segment known to be most affected in early-stage PD [190]. In contrast to SN NM volume, SN NM signal-to-noise ratio (SnR) is not consistently reduced in RBD patients [158, 184], suggesting that the SN NM volume is a better prodromal disease marker (at least at 3T). In the study by Pyatigorskaya, *et al.* [184], it was shown that combining SN NM volume and SnR differentiated the RBD patients from HC with 86% accuracy. The addition of SN fractional anisotropy further bolstered accuracy to 92%. In another study, Ehrminger, *et al*. [195] reported reduced NM SnR in the LC/sub-coeruleus complex of RBD patients, which is consistent with animal research showing that lesioning the LC/sub-coeruleus complex results in RBD [196]. Moreover, reduced LC/sub-coeruleus complex NM SnR differentiated patients from HC with 82.5% sensitivity and 81% specificity. Together, these findings highlight the potential of SN and LC/sub-coeruleus-focused NM-sensitive MRI for the identification of prodromal synucleinopathy. That said, a recent meta-analysis on the diagnostic value of MRI-measured SN NM in PD patients revealed substantial heterogeneity across the studies [197], presumably because of the various protocols and measurement techniques employed for data acquisition and analysis. Hence, the widespread use of NM-sensitive MRI is going to require the development and adoption of standardized approaches.

### 3.5 Diffusion MRI

In RBD patients, diffusion-weighted MRI has revealed diminished fractional anisotropy or axial diffusivity in several brainstem and midbrain regions, including the SN [184, 198, 199]. In contrast, Holtbernd *et al.* [200] reported increased FA in similar regions of RBD patients. Without FW correction, FA measures can be unreliable in tissues contaminated by inflammation or degeneration, and the two-compartment FW model can help in these instances [73, 82]. Recently, Zhou *et al.* [85] has shown that posterior SN FW levels are lowest in HC, intermediate in RBD patients, and highest in PD patients, consistent with the idea that PD-related nigral degeneration starts in the posterior SN [5, 8, 190] (Fig. **2C**). Posterior SN FW in the RBD patients was also inversely correlated with caudate DAT SPECT, suggesting that, like abnormal dopaminergic imaging [138], posterior SN FW may be a valuable indicator of prodromal disease. Despite the group-level differences reported by Zhou *et al.* [85], posterior SN FW only differentiated RBD patients from HC with moderate specificity (75%) and low sensitivity (62%), casting doubt that SN FW alone is a reliable prodromal marker. Other regions of the brain may be needed to bolster sensitivity and specificity.

### 3.6 Structural MRI

In RBD patients, voxel-based morphometry has revealed volume abnormalities, generally atrophy, in several cortical (*i.e.*, frontal cortex, cingulate cortex, parahippocampal gyrus, hippocampus, insula) and subcortical (*i.e.*, cerebellum, tegmental pons, putamen, caudate nucleus, thalamus) brain regions [198, 200-208]. Likewise, cortical thinning has been reported in all cortices of RBD patients [203-206, 209, 210], with cognitively impaired patients showing more extensive thinning [205]. Despite this, it is only recently that a link between RBD-related grey matter atrophy and subsequent phenoconversion has emerged. In a recently published prospective study, Pereira *et al.* [209] reported that cortical thinning of frontal, parietal, and occipital cortices were significant predictors of RBD phenoconversion over a ~3-year period (sensitivity: 95.2%; specificity, 100%) (Fig. **2D**). Although these results were obtained from only 27 RBD patients (6 of which were phenoconverted at follow-up), they suggest that cortical thinning is a promising marker of prodromal Parkinsonism.

### 3.7 Functional MRI

Several resting-state fMRI studies have revealed functional connectivity anomalies in RBD patients [153, 211-215]. Among these studies, those that enrolled both RBD and PD patients are arguably the most insightful because they allow functional connectivity patterns to be compared between prodromal and clinical Parkinsonism. In one of the studies, Ellmore *et al.* [212] reported that functional connectivity between the left SN and putamen significantly decreased from HC to RBD patients to PD patients, consistent with the idea that RBD patients are *en route* to an overt synucleinopathy. In a subsequent study, it was shown that compared to HC, RBD and PD patients show reduced resting-state functional connectivity within the basal ganglia and several cortical regions, most of which were part of the frontal lobes [213]. In that study, functional connectivity differentiated RBD patients from HC with high sensitivity (96%) and moderate specificity (78%). Because longitudinal/prospective studies have yet to be published, it remains unclear whether abnormal resting-state functional connectivity is a good predictor of short-term phenoconversion or not. Regardless, the evidence presented above suggests that fMRI can detect network anomalies associated with prodromal synucleinopathy.

To our knowledge, only one ASL study has investigated cerebral perfusion anomalies in RBD patients [216]. This study reported lower frontal cortex and insula CBF in RBD patients compared to healthy controls, which is consistent with perfusion SPECT findings [164, 166-168]. Given that ASL-based CBF is a proxy of metabolic activity [109, 110], it could potentially be used to identify metabolic covariance patterns that may help predict phenoconversion (*e.g.*, [159, 160, 162]). Should this be the case, ASL could be a valuable alternative to both ^18^F-FDG PET and perfusion NI going forward.

### 3.8 Interim Summary

Numerous dopaminergic and metabolic imaging studies have reported anomalies in RBD patients, highlighting the usefulness of NI for the identification of prodromal synucleinopathies. Some of these studies further suggest that dopaminergic and metabolic imaging can be used to predict short-term conversion to an overt synucleinopathy, which could help select candidates for clinical trials. The fact that an abnormal dopaminergic PET/SPECT scan is a several-fold stronger indicator of prodromal PD than most clinical markers (including an abnormal UPDRS/MDS-UPDRS score) further underscores the value of dopaminergic imaging as a prodromal disease biomarker. Iron-sensitive, NM-sensitive, diffusion-weighted, structural, and functional MRI studies have also revealed differences between RBD patients and HC. These differences, however, need to be replicated. Moreover, given the paucity of longitudinal MRI studies in RBD patients, it remains unclear whether MRI is as effective as dopaminergic imaging in terms of predicting short-term phenoconversion. Ongoing research on RBD patients will help establish where different MRI biomarkers stand, compared to NI and clinical markers, as indicators of prodromal synucleinopathy.

## 4 BIOMARKERS FOR THE PROGRESSION OF PARKINSONIAN DISORDERS

RBD patients are the ideal candidates for neuroprotective trials aimed at slowing/stopping the development of synucleinopathies because most patients will convert to PD, MSA, or DLB within a decade [140, 141]. Ideally, the clinical endpoint of these trials would be phenoconversion because the goal is to slow/stop the emergence of overt synucleinopathy. Unfortunately, predicting the short-term phenoconversion of RBD patients come with considerable uncertainty [140, 141], even when these predictions are informed by dopaminergic imaging [144]. Similar issues plague clinical trials targeting patients with overt Parkinsonian disorders. In these trials, outcome variables would ideally be obtained from meaningful clinical scales, which remain the gold standard for evaluating disease severity. As mentioned in the introduction of this review, however, symptom-based scales cannot distinguish between disease-modifying and symptomatic intervention effects. They are also subjective and confounded by interrater variability, which can mask short-term therapeutic benefits. Although this may not be critical for clinical trials targeting quickly progressing Parkinsonian disorders such as MSA, PSP, and CBD, it may be for trials targeting PD, which progresses more slowly. One way to circumvent these issues is to use objective and quantitative progression biomarkers that predict phenoconversion and long-term disease progression.

### 4.1 Dopaminergic Imaging

**Fig. (3):**
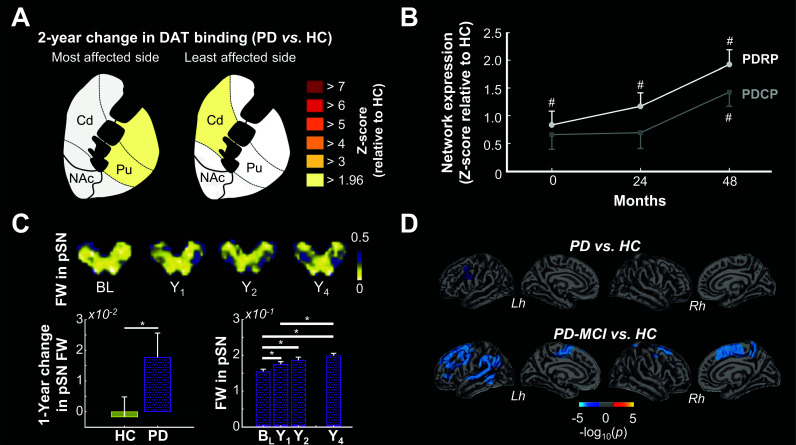
(**A**) Longitudinal (2-year) reductions in striatal DAT binding in PD patients *vs.* HC (adapted from Biondetti *et al.* [158]). (**B**) 4-year changes in PDRP and PDCP expression in PD patients (adapted from Huang *et al.* [226]). (**C**) Axial FW image showing increasing pSN FW in a single PD patient followed over 4 years (top). 1-year change in pSN FW in HC and PD (bottom left) and 4-year change in pSN FW in PD (bottom right) (adapted from Burciu *et al.* [79]). (**D**) Vertex-wise comparisons of cortical thinning in PD (top) and PD-MCI (bottom) compared to HC over 18 months (adapted from Mak *et al.* [256]). **Abbreviations**: BL, baseline; RBD, rapid eye movement behaviour disorder; PD, Parkinson’s disease; Cd, caudate nucleus; NAc, nucleus accumbens; Pu, Putamen; HC, healthy controls; PDRP, Parkinson’s disease-related pattern; PDCP, Parkinson’s disease cognitive pattern; pSN, posterior substantia nigra; FW, free-water; Y1, Year 1; Y2, Year 2; Y4, Year 4; PPMI, Parkinson’s Progressive Marker Initiative; PD-MCI, Parkinson’s disease with mild cognitive impairment; Lh, left hemisphere; Rh, right hemisphere; #, *p* < 0.05 *vs*. HC at baseline; *p < 0.05.

Longitudinal studies have reported steeper decrements in striatal DAT binding in RBD patients compared to HC [146, 158] (Fig. **3A**). Likewise, striatal F-Dopa [217, 218], DAT [158, 218-223] and VMAT2 [218] uptake/binding values have been shown to diminish longitudinally in early-stage PD patients and atypical Parkinsonism, with the latter generally presenting with faster decline rates. Together, these findings suggest that dopaminergic imaging could potentially track progression in both prodromal and manifest Parkinsonism. However, there are potential caveats to the assessment of disease progression with dopaminergic imaging. First, some research suggest that nigrostriatal degeneration slows as Parkinsonian disorders develop [217, 218, 223-225]. It follows that dopaminergic imaging may lose its ability to track disease progression *as* the disease progresses. In fact, neuropathological research has shown that striatal tyrosine hydroxylase and DAT staining are drastically reduced within 4-5 years of PD onset [8], meaning that dopaminergic imaging is less effective for tracking late-stage PD progression. A second caveat is that PD-related longitudinal changes in nigrostriatal dopaminergic function have generally not been related to changes in clinical function [217, 219, 220, 225, 226], which raises some concerns about its value as a disease progression biomarker. Finally, dopaminergic imaging is sensitive to disease-independent pharmacological effects [227, 228].

### 4.2 Metabolic Imaging

If cross-sectional studies that enrolled both RBD and PD patients are any indication, ^18^F-FDG PET-derived covariance patterns could be used to track prodromal disease progression. Indeed, studies have shown that PDRP expression in RBD patients generally lies between that of HC and PD patients [151, 161], suggesting that the PDRP should increase with disease progression. In line with this, Kogan *et al.* [162] reported a significant increase in PDRP expression in RBD patients over four years, with all patients showing higher scores at follow-up than at baseline. These authors also found a positive correlation between changes in PDRP z-scores and changes in UPDRS motor ratings per year, suggesting that longitudinal changes in the PDRP track prodromal disease severity. In early-stage PD patients, Huang *et al.* [226] reported that both the PDRP and its cognitive counterpart, the PD-related cognitive pattern (PDCP), increase significantly over four years (Fig. **3B**). Despite not having a longitudinal control group, this study is worth highlighting for two reasons. First, the longitudinal PDRP changes positively correlated with concomitant changes in UPDRS motor ratings, confirming the potential value of the PDRP as a proxy for disease progression. Second, PDCP increases lagged behind those of the PDRP, raising the possibility that together, these covariance patterns could track PD progression from early to late disease stages. More longitudinal work is needed to confirm this, especially over a shorter timeline.

Only a few studies have investigated how the progression of atypical Parkinsonian disorders alters brain metabolism. Lyoo *et al.* [229] compared ^18^F-FDG PET measurements from MSA patients with different disease durations with those of HC. They reported that early-stage MSA (disease duration ≤ 1 year) was the associated is with frontal cortex and cerebellar hypometabolism, whereas in later-stage MSA (disease duration 1-3 years), hypometabolism further extended to the parietal and temporal cortices, as well as the striatum. In one of the few longitudinal ^18^F-FDG PET studies on MSA patients, Lee *et al.* [230] investigated the effects of mesenchymal stem cell therapy on disease severity and brain metabolism over 12 months. In the control MSA group, the authors reported longitudinal metabolism decrements in frontal, cingulate, cerebellar, and brainstem areas, which were accompanied by increases in UMSARS scores. In the MSA patients who received mesenchymal stem cell therapy, the authors reported longitudinal metabolism increases in frontal, temporal, and cerebellar areas, which were accompanied by decreases in UMSARS scores. Although no correlational analyses were carried out between longitudinal changes in ^18^F-FDG uptake and UMSARS ratings, the opposite trends between these variables suggest that the broadening and worsening of glucose hypometabolism is an indicator of MSA progression. Whether ^18^F-FDG PET can track the progression of Parkinsonian tauopathies currently remains unclear.

### 4.3 𝛼Syn and Tau Nuclear Imaging

𝛼Syn imaging could potentially be used to track the progression of Parkinsonian synucleinopathies. However, 𝛼Syn imaging is not yet feasible *in vivo*. Developing an 𝛼Syn tracer is challenging for several reasons. First, Lewy pathology is largely an intracellular phenomenon, thus, candidate compounds must be small and lipophilic [231]. Second, there are structural differences between the recombinant 𝛼Syn fibrils often used to select PET compounds and the 𝛼Syn fibrils found in post-mortem PD, MSA, and DLB brains [232, 233]. Although recombinant fibrils are indispensable given the limited availability of patient fibrils, it is possible that compounds selected for their affinity with recombinant fibrils will not show commensurate affinity with patient fibrils [231]. Third, because 𝛼Syn fibrils are structurally similar to, and less abundant than, beta-amyloid and tau fibrils, candidate ligands need to show remarkable target affinity and specificity to mitigate off-target binding. Despite these hurdles, immense progress has been made in recent years. For instance, Kuebler *et al.* [234] developed a PET tracer ([^3^H]MODAG-001) based on the structure of diphenyl-pyrazol, which has been demonstrated to bind with 𝛼Syn [235] and reduce its pathological aggregation [236]. *In vitro*, [^3^H]MODAG-001 showed sub-nanomolar affinity (K_d_ = 0.6 nM) with recombinant 𝛼Syn fibrils, which was ~30 times greater than its affinity with either beta-amyloid or tau fibrils. In mice injected with recombinant 𝛼Syn, C^11^[^3^H]MODAG-001 showed evidence of binding in the striatum, suggesting that it both passes the blood-brain barrier and finds its way to the cytosol. Although these findings are encouraging, [^3^H]MODAG-001 autoradiography failed to detect 𝛼Syn binding in DLB brain homogenate, probably as a consequence of excessive off-target binding with background proteins and/or differences between recombinant and disease-specific 𝛼Syn.

Unlike 𝛼Syn imaging, tau imaging is readily available [237]. However, one of the study suggests it is inferior to structural MRI when it comes to tracking PSP progression [238]. Despite this, it is reasonable to believe that tau imaging could reliably track the progression of Parkinsonian tauopathies if certain shortcomings, namely off-target binding and tau isoform specificity, are addressed. Concerning off-target binding, Lowe *et al.* [239] conducted an autoradiographic study using the tau ligand flortaucipir (AV-1451) and reported abundant binding in blood vessels, the SN, high iron areas, the choroid plexus, and the melanin found between the arachnoid and pia mater. Tau ligands have also been shown to have a high affinity with neuromelanin, monoamine oxidase A/B, and other unidentified molecules [240]. Indeed, studies have shown that ^18^F-AV-1451 binding is reduced in the SN of PD and PSP patients [241, 242], consistent with the fact that nigral neuromelanin diminishes with DA cell loss [187]. Concerning tau isoform specificity, most tau ligands were initially developed to target the tau fibrils present in Alzheimer’s disease (*i.e.*, paired helical filaments) [243], which differ from those found in Parkinsonian tauopathies (4-repeat tau fibrils) [244, 245]. Although pyridinyl-butadienyl-benzothiazole 3 (PBB3) has shown affinity with both paired helical and 4-repeat tau fibrils [246], ligands that specifically bind with the latter are required to reliably monitor the progression of Parkinsonian tauopathies. If the immense progress made in recent years is an indication of things to come, tau and 𝛼Syn PET tracers that can reliably track disease are on the horizon.

### 4.4 NM-sensitive MRI

NM-sensitive MRI may track PD progression depending on what measurement is used. For instance, longitudinal (~2-year) SN NM *SnR* changes do not differ between RBD patients, early- and late-stage PD patients, and HC [158, 247]. In contrast, the SN NM volume/area diminishes longitudinally in both early- and late-stage PD [247, 248], suggesting that morphological SN NM measurements can track progression in manifest PD. Gaurav *et al.* [247] estimated that ~40-60 participants (per arm) would be required to detect a 50% reduction in the rate of SN NM volume change in a 1-year clinical trial. This estimate, however, is difficult to conclude because the study was performed over two years, and thus estimating a 1-year change based on 2-year data is based on assumptions. Moreover, Gaurav *et al.* [247] did not find a relationship between longitudinal SN NM volume changes and clinical function, perhaps because of the low resolution of NM-sensitive MRI at 3T. Future work with stronger MRI systems is warranted to confirm the value of NM-sensitive MRI for tracking PD progression and to investigate whether this technique can track progression in atypical Parkinsonism whether or not.

### 4.5 Diffusion MRI

In recent years, it has become clear that nigral FW is a promising measurement for PD progression. For instance, FW increases significantly in the posterior, but not anterior, SN of RBD patients over a ~2-3-year period compared to HC [85]. Likewise, single- and multi-site cohort studies have shown that posterior SN FW increases longitudinally in early-stage PD patients [79, 83, 249] (Fig. **3C**). These studies also showed that posterior SN FW positively correlates with 1-year changes in bradykinesia [83, 249], MDS-UPDRS motor scores [249] and that one- and two-year changes in posterior SN FW predicts the four-year progression on the Hoehn and Yahr staging system [79]. Together, these findings indicate that nigral FW assessments can predict early-stage PD progression. Although anterior SN FW does not increase longitudinally in prodromal [85] or early-stage PD [79, 83], it increases longitudinally in late-stage PD (disease duration: ~7 years) [80], suggesting that FW imaging can track the well-established caudo-rostral pattern of nigral degeneration in PD [250]. Burciu *et al.* [79] estimated that ~65-100 participants (per arm) would be required to detect a 50% reduction in the rate at which the posterior SN accrues FW in a 2-year clinical trial in early-stage PD. In comparison, almost twice as many participants would be required to detect a commensurate effect using DAT SPECT [79]. Moreover, it has been demonstrated that nigrostriatal FW and FA_T_ are not acutely affected by antiparkinsonian drugs [251], which makes diffusion MRI attractive for tracking disease progression in clinical trials. Additional longitudinal research is needed to confirm the value of SN FW imaging for tracking disease progression in prodromal and late-stage PD.

Longitudinal diffusion MRI studies have reported several abnormal microstructural changes in MSA, PSP, and/or CBD [252-254]. Some of these studies also found correlations between various diffusion MRI metrics and longitudinal changes in cognitive, motor, and/or ocular function in these patient populations [253, 254]. However, these studies did not account for neurodegeneration-related increased FW. Hence although their results suggest progressive microstructural changes in atypical Parkinsonian disorders, it is difficult to determine the biological nature of these changes and whether they can be used as robust progression makers. More longitudinal research is needed to confirm the usefulness of diffusion MRI, and specifically FW imaging, for tracking disease progression in atypical Parkinsonian disorders.

### 4.6 Structural MRI

Evidence suggests that structural MRI can track progression in prodromal and overt Parkinsonian disorders. Campabadal *et al.* [210] reported greater rates of parietal, frontal, and occipital cortex thinning over ~1.6 years in RBD patients compared to HC, with parieto-frontal thinning correlating with changes in UPDRS-III scores. Likewise, longitudinal (~19-48 months) structural MRI studies have generally reported faster rates of cortical thinning, most often in the frontal lobe, in early- to moderate-stage PD patients compared with HC [255-258] (although see [259]), with cognitively impaired patients showing more extensive thinning (Fig. **3D**). In one of these studies, changes in cortical thickness positively correlated with changes in Montreal Cognitive Assessment (MoCA) scores [255], suggesting a link between cortical thinning and cognitive decline in PD. In another study, Sterling *et al.* [260] investigated longitudinal cortical gyrification changes in early- (< 1 year), moderate- (1-5 years), and late-stage (> 5 years) PD patients over 36 months. Cross-sectionally, gyrification was inversely correlated with UPDRS motor scores. Longitudinally, gyrification loss was significantly greater in moderate-stage PD and tended to be greater in early-stage PD, compared to HC. In the late-stage PD group, gyrification loss was not different from HC. Overall, these results suggest that structural MRI can track disease progression in prodromal as well as early- to moderate-stage PD.

Structural MRI metrics can also be used to track longitudinal atrophy changes in PSP, MSA-P, and CBD [261-265], with atrophy often correlating with the changes in clinical function [261, 263, 264]. Recently, Quattrone *et al.* [266] have shown that the MRPI can track progression in PSP. Specifically, these authors estimated that ~45-85 participants (per arm) would be required to detect a 40% reduction in the rate of MRPI progression in a 2-year clinical trial (depending on whether PSP-RS or PSP-P are enrolled).

### 4.7 Interim Summary

Both NI and MRI have shown potential for tracking the progression of Parkinsonian disorders, making them interesting outcome variables for clinical trials. Compared with NI, MRI can considerably reduce research-related costs, especially if a clinical trial requires multiple assessments of disease progression.

## CONCLUSION

From the reviewed literature, we conclude that MRI can assist with the differential diagnosis of Parkinsonian disorders, identify anomalies in prodromal Parkinsonism, and track disease progression at least as well as NI. The advantage of NI over MRI is that dopaminergic and metabolic imaging can predict short-term phenoconversion in prodromal Parkinsonism. MRI may also be able to do this (*e.g.*, Pereira *et al.* [209]) but more longitudinal/prospective MRI studies in prodromal Parkinsonism are needed to confirm it. Notwithstanding, MRI may be preferred over NI because it is less expensive, more accessible, and non-dependent on ionizing radiation.

So, where should we go from here? Restricting ourselves to the scope of this review, we believe that more prospective research is needed to firmly establish and confirm the value of MRI for monitoring prodromal disease and predicting phenoconversion. Promising results across most MRI modalities also need to be replicated in large, multi-center cohorts to establish robustness. As high-field imaging becomes a commodity, it will be interesting to see whether the different MRI markers discussed in this review can further improve differential diagnosis, prodromal disease identification, and disease monitoring. Although NI can complement or be a substitute for MRI in all the areas covered in this review, we believe that its most meaningful impact will come once reliable 𝛼Syn and tau tracers are available. Indeed, if Parkinsonian proteinopathy tracers prove to be potent biomarkers for therapeutic discovery, NI may become an indispensable tool in the quest for efficient disease-modifying therapies.

## LIST OF ABBREVIATIONS

𝛼Syn: Alpha-synuclein
AID-P: Automated Imaging Differentiation in Parkinsonism
ASL: Arterial Spin Labelling
BOLD: Blood-oxygen-level-dependent
CBD: Corticobasal Degeneration
CBF: Cerebral Blood Flow
CBFS: Cortical Basal ganglia Functional Scale
CC: Corpus Callosum
DA: Dopamine
DAT: Dopamine Transporter
DLB: Dementia with Lewy Bodies
EMG: Electromyography
FA_T_: Fractional Anisotropy
F-Dopa: Fluorodopa
fMRI: Functional Magnetic Resonance Imaging
FW: Free-water
^18^F-AV-1451: Flortaucipir-^18^F
^18^F-FDG: ^18^F-fluorodeoxyglucose
HC: Healthy Controls
LC: Locus Coeruleus
MDS-UPDRS: Movement Disorders Society's Unified Parkinson's Disease Rating Scale
MoCA: Montreal Cognitive Assessment
MRI: Magnetic Resonance Imaging
MRPI: Magnetic Resonance Parkinsonism Index
MSA: Multiple System Atrophy
MSA-C: Multiple System Atrophy-Cerebellar Type
MSA-P: Multiple System Atrophy-Parkinsonian Type
NI: Nuclear Imaging
NM: Neuromelanin
PBB3: Pyridinyl-butadienyl-benzothiazole 3
PCA: Principal Component Analysis
PD: Parkinson’s Disease
PDCP: Parkinson’s Disease-related Cognitive Pattern
PDRP: Parkinson’s Disease-related Pattern
PET: Positron Emission Tomography
PSP: Progressive Supranuclear Palsy
PSP-P: Progressive Supranuclear Palsy Parkinsonism
PSP-RS: Progressive Supranuclear Palsy Richardson’s Syndrome
PSPRS: Progressive Supranuclear Palsy Rating Scale
QSM: Quantitative Susceptibility Mapping
RBD: Rapid-eye-movement Behavior Disorder
SN: Substantia Nigra
SnR: Signal-to-noise Ratio
SPECT: Single-photon Emission Computed Tomography
SVM: Support Vector Machine
SWI: Susceptibility-weighted Imaging
UMSARS: Unified Multiple System Atrophy Rating Scale
VMAT2: Vesicular Monoamine Transporter 2
